# Supplementary material for: Generative prediction of real-world prevalent SARS-CoV-2 mutation with in silico virus evolution
Source: Brief Bioinform. 2025 Jun 18;26(3):bbaf276. doi: 10.1093/bib/bbaf276 (PMC12204194; doi:10.1093/bib/bbaf276)
Supplement: Supplementary_material_bbaf276 [file supplementary_material_bbaf276.pdf]

# Contents


S1 Dataset selection and preprocessing . . . . . 2
S2 Pretrained model selection . . . . . 2
S3 Protein language model fine-tuning . . . . . 3
S4 Variant generation . . . . . 3
S5 Implementation of the screening models . . . . . 4
S6 Mutation ranking criterion . . . . . 5
S7 Ablation experiments for variant generation scale . . . . . 5
S8 The remaining number of variants at different screening stages . . . . . 6
S9 The ranking trends of previous real-world prevalent mutations across different screening stages . . . . . 7
S10 Ablation experiments for core modules . . . . . 7
S11 Comparison with MLAEP . . . . . 8
S12 The effects of introducing binding affinity prediction model . . . . . 10
S13 Reconstruction of viral evolution to the Omicron lineage . . . . . 11
S14 Recommendation pipeline of future prevalent mutations . . . . . 11
S15 *In vitro* experimental validation . . . . . 12
S16 Ethical guidelines and policy implications . . . . . 13

# Supplementary information

## S1 Dataset selection and preprocessing

First, the sequence set for PLM fine-tuning and variant generation were integrated from the GISAID database. Specifically, for the ablation experiments in Section 2.2 and the *in silico* validation experiments in Section 2.3, RBD sequences of variants between the starting and the cutoff lineages were integrated into a sequence set for PLM fine-tuning and calculation of mutated probabilities of all sites. In addition, for the predictive experiments in Section 2.4, RBD sequences of variants that appeared after the starting lineage were integrated into a sequence set for the PLM fine-tuning and calculation of mutated probabilities of all sites.

Second, the deep mutational scanning (DMS) datasets of previous work [1] were adopted to train a host-level expression prediction model. There were seven groups of DMS datasets including wild type, Alpha, Beta, Delta, Eta, Omicron BA.1 and Omicron BA.2. We only used the samples of Omicron BA.1 and Omicron BA.2 and normalized the RBD expression labels with the value of Omicron BA.2 to get final labels for training. Positive samples were defined as those with labels greater than 1, while others were negative samples. Finally, there were 7,182 negative samples and 639 positive samples in the training dataset.

Third, two sets of DMS datasets from previous studies [2, 3] were adopted to build the antibody barrier models. There were high-throughput mutation escape profiles for thousands of antibodies in the above DMS datasets, including mutations, escape scores, antibody names, and the group that each antibody belongs to. In the work of [2], the antibodies were isolated from the convalescents of SARS, SARS-CoV-2 Wild-type, BA.1, BA.2 and BA.5 variants. In the work of [3], the antibodies were isolated from the convalescents of BA.1, BA.2, BA.5 and BF.7 variants, the reinfection convalescents of BA.1, BA.2 variants and long-term BA.1 convalescents. In this work, we only used the DMS data of antibodies isolated from convalescents of Omicron variants.

## S2 Pretrained model selection

In this work, we adopted a protein language model ESM-2 instead of AlphaFold3 [4] or ESM3 [5] for mutant generation and expression prediction task due to its suitability for SARS-CoV-2 evolution prediction. First, AlphaFold3 is a protein structure prediction model and struggles to deal with the viral protein sequences with a few mutations [6–8] Even if the feature extraction module of AlphaFold3 might be able to extract features for virus property prediction, the mutated sequences in the deep mutational scanning data of SARS-CoV-2 used in this work are very similar to each other (usually 1-2 different residues), making the predicted structures indistinguishable from each other, thereby affecting the prediction performance. Second, ESM3 is a multi-modal protein language model, which takes protein sequences, property description texts and protein structures as input rather than sequence itself. It emphasizes more on multi-modal capabilities, while ESM-2 only takes protein sequences as input, and only adopts masked residue prediction as pretraining task. In the scenario of SARS-CoV-2 evolution prediction, the property description text and structure information are

unnecessary. Therefore, ESM-2 is more suitable for fitting the distribution of given variant sequences and generating new variants that conform to the distribution.

## S3 Protein language model fine-tuning

For protein language model fine-tuning, we employ the real-time evolutionary trajectory information of the starting lineage (the reference sequence of *in silico* evolution) as the future mutational pattern, where all variant sequences (SARS-CoV-2 RBDs here) that appears after the starting lineage from GISAID database [9] are integrated into a sequence set. Subsequently, the real-time virus evolutionary information of the starting lineage carried by the above sequence set is captured through unsupervised fine-tuning to adjust the initially learned mapping of position-by-position residue type distributions.

Specifically, for a given starting lineage $x_s$ with length $L$, we collected variant sequences emerging after $x_s$ (also before the cutoff lineage $x_t$ of the ablation experiments in Section 2.2 and *in silico* validation experiments in Section 2.3) to build a sequence set $F$. The above integrated sequence set $F$ was used to fine-tune the pretrained PLM with a masked residue prediction task. The widely recognized ESM-2 (650M) was adopted in this work, which can be replaced by any powerful pretrained PLM, such as ProtTrans [10] and ProteinBERT [11]. To balance the novelty and diversity of the generated variant sequences, we set the fine-tuning epoch as 1 epoch so that the PLM was trained on all the collected variant sequences while preserving the diversity of generation.

## S4 Variant generation

The core of variant generation lies in two points: where to mutate (determine the site to be mutated) and how to mutate (determine the mutation type at that site). First, we calculated the mutated probability of each site $P$ using the sequences in $F$ as

$$P_i = \frac{1}{|F|}\sum_f (1 - \mathbb{I}(f[i] == x_s[i])), f \in F, i \in [1, L], \tag{1}$$

where $|F|$ denotes the number of sequences in $F$, and $\mathbb{I}(x) = 1$ if $x$ is $true$, otherwise $\mathbb{I}(x) = 0$.

Second, with the calculated $P$ and the fine-tuned PLM, we conducted the variant sequence generation through a mutation process based on the starting lineage $x_s$. Each site $i$ of $x_s$ performed a binomial trial with the success probability of $P_i$, i.e. $p_i \sim B(P_i)$, and the site $i$ was updated as

$$x_s^{'}[i] = p_i \cdot [MASK] + (1 - p_i) \cdot x_s[i], p_i \in \{0, 1\}, \tag{2}$$

where $x_s[i]$ denotes the $i$-th residue of the sequence and $[MASK]$ denotes the mask token. The equation means that each residue $i$ has the probability of $P_i$ to be replaced by mask token. We rejected the $x_s^{'}$ with mask token number greater than $MAX_{mask}$. In our experiments, $MAX_{mask}$ was set as 5.

Third, the fine-tuned PLM FT was used to complete the masked sequences following the adjusted residue distribution as

$$\hat{x}_s[i] = \mathrm{FT}(x_s^{'})[i], i \in \{i | x_s^{'}[i] = [MASK]\}. \quad (3)$$

The above generation process was repeated until the generated variant sequences reach the generation scale $S$.

## S5 Implementation of the screening models

The implementation details of host-level expression prediction model are as follows. First, for sequence embedding extraction, the off-the-shelf pretrained PLM was adopted to extract amino-acid-level embeddings of mutated RBD sequences. Second, the CNN and ContextPool-attention [12] were adopted to capture the local and global dependencies of the mutations respectively. The one-dimension CNN adopted here was to build the impact of a mutation on their sequentially neighboring residues, i.e., the local dependencies of the mutation. It consisted of 3 convolution layers with Layer Normalization and used leaky rectified linear unit (Leaky ReLU) as activation function. Correspondingly, the ContextPool-attention with dynamic granularity was adopted here to build the motif-level global dependencies of a mutation. The Gaussian mask vector was normalized with its maximum value during calculation for stable training. The above local feature and motif-level global feature of mutations were subsequently concatenated and max-pooled on the residue dimension to obtain the protein-level embeddings. Third, the model was trained through multi-task learning of a binary classification task (expression increases or not) and a regression task (expression strength estimation), where a multi-task focal loss was adopted to alleviate the imbalance between positive and negative samples.

The implementation details of herd-level quantified antibody barrier model are as follows. First, the DMS datasets from the previous studies [2, 3] were adopted to build the quantified antibody barrier model. Considering the differences in the neutralization patterns of antibodies in different groups, we calculated the average escape score in each antibody group for each mutation. Specifically, for the antibody group $G$, we defined $|G|$ as the number of antibodies of $G$. For the mutation $(s, m)$, where $s$ denotes mutated site and $m$ denotes the residue mutated to, we calculated the average of all the escape scores $i$ of this mutation in group $G$, i.e.

$$f_G(s, m) = \frac{1}{|G|} \sum_{site_i = s,\ mut_i = m} score_i, \quad (4)$$

where $site$ denotes the mutated site, $mut$ denotes the residue type and $score$ denotes the escape score. Here, we can get a function for each group that maps mutation to the average escape score of the group, i.e.

$$f_G : (s, m) \to score_G. \quad (5)$$

Second, for a generated variant (i.e. mutated RBD), it may contain many mutations compared with wild-type RBD sequence, which can be denoted as $\{(s_1, m_1), (s_2, m_2), ..., (s_n, m_n)\}$. Based on the mapping function above, we summed the average escape scores of all the mutations as the group escape score of the variant for each antibody group. For the antibody group $G$, the group escape score of a variant was calculated as

$$F_G = \sum_{i=1}^{n} f_G(s_i, m_i). \tag{6}$$

Third, the group escape scores of different antibody groups were weighted summed based on the importance of each antibody group to get the final herd escape score $Barrier$, i.e.

$$Barrier = \sum_{G} w_G F_G, \tag{7}$$

where $w_G$ denotes the weight of antibody group $G$. Here, we used the proportion of antibodies in each group as weight.

## S6 Mutation ranking criterion

For the prediction results of host-level expression prediction model, we selected the variants with enhanced expression level compared to BA.2, i.e., the variants with output probabilities of classification prediction larger than 0.5 were retained. Subsequently, for the prediction results of herd-level quantified antibody barrier model, we sorted all the variants with herd escape score, and selected top 50% variants with relatively high escaping capability against population. Finally, the frequency of each mutation in the screened variant sequences was calculated for ranking, where the top-ranked mutations were considered to have the potential to become prevalent in the future. It should be noted that two sets of DMS datasets from the previous studies [2, 3] were adopted to build two types of quantified antibody barrier models, and two rankings for the same mutation from the above two types of quantified antibody barrier models were averaged to mitigate the impact of experimental data bias to obtain the final ranking result.

## S7 Ablation experiments for variant generation scale

As shown in Fig.3a, multiple sets of generation experiments with an interval of 50,000 were performed to explore whether the escape capability increment approaches 0 under two types of quantified antibody barrier models. The escape capability increment here is the difference in the average herd escape score of the top $K$ variants sorted by scores in two consecutive generation experiments. We continued to expand the generation scale from an initial scale of 50,000 and observe the downward trend of escape capability increment under different $K$ values, including 5,000, 10,000, 15,000, and 20,000. The data of ablation experiments for variant generation scale under two types of quantified antibody barrier models are stored in two separate files (antibody_barrier_model_I.csv, antibody_barrier_model_II.csv) at https://github.com/Kevinatil/ViralForesight.

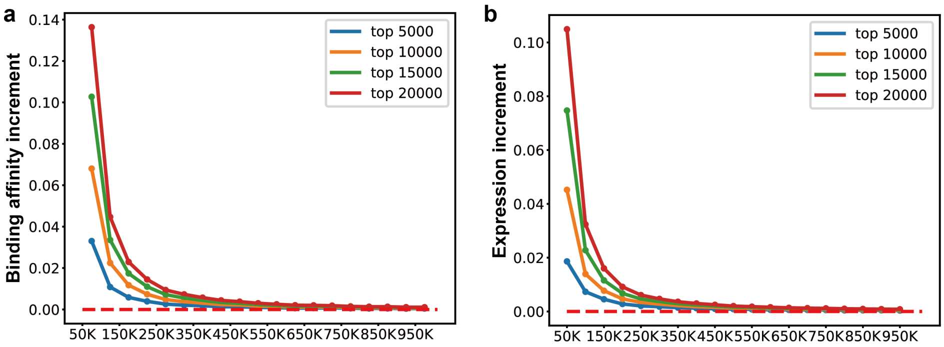


**Fig. S1** Ablation experiments for variant generation scale of binding affinity increment (a) and expression increment (b).

# S8 The remaining number of variants at different screening stages

| Repeat experiment | Stage I | Stage II | Stage III |
|---|---|---|---|
| Experiment 1 | 1,000,000 | 373,201 | 213,784 |
| Experiment 2 | 1,000,000 | 373,313 | 214,045 |
| Experiment 3 | 1,000,000 | 373,418 | 213,597 |

**Table S1** The remaining number of variants at different stages under quantified antibody barrier model I

| Repeat experiment | Stage I | Stage II | Stage III |
|---|---|---|---|
| Experiment 1 | 1,000,000 | 373,201 | 179,474 |
| Experiment 2 | 1,000,000 | 373,313 | 179,519 |
| Experiment 3 | 1,000,000 | 373,418 | 179,477 |

**Table S2** The remaining number of variants at different stages under quantified antibody barrier model II

## S9 The ranking trends of previous real-world prevalent mutations across different screening stages

| Mutation | stage I | stage II | stage III |
|---|---|---|---|
| L452R | 6 | 2 | 2 |
| L452Q | 5 | 1 | 3 |
| R346T | 54 | 22 | 15 |
| F486S | 81 | 28 | 20 |
| F486P | 84 | 35 | 26 |
| F486V | 83 | 49 | 47 |
| F490S | 150 | 70 | 55 |
| K444T | 110 | 122 | 68 |

**Table S3** The ranking trends of previous real-world prevalent mutations across different screening stages with BA.2.1 as the starting lineage

| Mutation | stage I | stage II | stage III |
|---|---|---|---|
| R346T | 7 | 4 | 3 |
| R346S | 2 | 5 | 4 |
| K444T | 84 | 69 | 36 |
| G446R | 43 | 37 | 24 |
| F490S | 70 | 41 | 23 |
| N450D | 131 | 95 | 75 |

**Table S4** The ranking trends of previous real-world prevalent mutations across different screening stages with BA.5.1 as the starting lineage

## S10 Ablation experiments for core modules

In this section, we conducted in-depth ablation analysis on the core modules in ViralForesight. First, we performed ablation experiments on PLM fine-tuning with BA.2.1 as the starting lineage. As shown in Table.S5, we found that 3 out of the 14 target mutations did not appear, which was most likely due to the fact that without PLM fine-tuning results in insufficient generation of potential high-risk sequences. At the same time, the remaining 11 target mutations that appeared were all ranked significantly lower. Second, we performed ablation experiments on mutated probability calculation with BA.2.1 as the starting lineage. As shown in Table.S6, when we did not calculate the mutated probability of each site when generating variants, the rankings of all 14 target mutations decreased significantly. Third, the effect of quantified antibody barrier model was demonstrated by the experiments in Fig.3d, Table.S3, and Table.S4. "Stage III" referred to the stage that the variants were further screened by

the quantified antibody barrier model, and we found that most of the target mutations were ranked higher in Stage III. Overall, the above ablation results demonstrated that the core modules of ViralForesight are beneficial to its performance in predicting the real-world prevalent SARS-CoV-2 mutations.

| Mutation | w/ PLM fine-tuning | w/o PLM fine-tuning |
|---|---|---|
| 452Q | 1 | - |
| 452R | 2 | 77 |
| 346T | 15 | 32 |
| 486S | 22 | 39 |
| 460K | 25 | 64 |
| 486P | 26 | 298 |
| 446S | 37 | 53 |
| 486V | 47 | 62 |
| 490S | 55 | 75 |
| 339H | 62 | - |
| 444T | 67 | 83 |
| 445P | 116 | 294 |
| 368I | 141 | 179 |
| 478R | 188 | - |

**Table S5** Ablation experiments on PLM fine-tuning with BA.2.1 as the starting lineage

| Mutation | w/ mutated probability calculation | w/o mutated probability calculation |
|---|---|---|
| 452Q | 1 | 11 |
| 452R | 2 | 27 |
| 346T | 15 | 102 |
| 486S | 22 | 32 |
| 460K | 25 | 100 |
| 486P | 26 | 43 |
| 446S | 37 | 222 |
| 486V | 47 | 98 |
| 490S | 55 | 63 |
| 339H | 62 | 989 |
| 444T | 67 | 129 |
| 445P | 116 | 147 |
| 368I | 141 | 167 |
| 478R | 188 | 1186 |

**Table S6** Ablation experiments on mutated probability calculation with BA.2.1 as the starting lineage

## S11 Comparison with MLAEP

MLAEP [13] leveraged binding specificity prediction model and genetic algorithm to generate sequences with higher binding specificities. Although MLAEP can predict specific mutations, it performed worse than ViralForesight in prediction quantity and

| Mutation | ViralForesight | MLAEP |
|---|---|---|
| 452R | 1 | 59 |
| 452Q | 3 | 73 |
| 346T | 8 | 22 |
| 486S | 9 | 2,397 |
| 486P | 13 | 2,768 |
| 460K | 27 | 1,366 |
| 486V | 32 | 2,376 |
| 446S | 35 | 71 |
| 490S | 40 | 76 |
| 339H | 62 | 218 |
| 444T | 73 | 851 |

**Table S7** Ranking improvement of correctly predicted previous real-world prevalent mutations with BA.2.1 as the starting lineage

| Mutation | ViralForesight | MLAEP |
|---|---|---|
| 346T | 2 | 2 |
| 346S | 3 | 3,606 |
| 490S | 22 | 3,615 |
| 446R | 23 | 3,364 |
| 460K | 31 | 276 |
| 444T | 35 | 2,578 |
| 450D | 74 | 341 |

**Table S8** Ranking improvement of correctly predicted previous real-world prevalent mutations with BA.5.1 as the starting lineage

ranking performance, as shown in Fig.4. More importantly, we found that its prediction would convergent to certain sites when the variety of the initial sequence set is low. As shown in Fig.S2, when we adopted BA.2.1 as starting sequence, and only used Omicron variants before BA.2.1 as initial sequence set, the predicted mutation sites only included 346 and 490. However, with only Omicron variants for PLM fine-tuning, ViralForsight predicted more potential mutation sites, including 339, 346, 444, 446, 452, 460 and 486. Overall, the pretrained PLM is more suitable for variant generation by capturing protein evolution patterns [14], so that ViralForesight can predict potential mutation types even with limited available lineages.

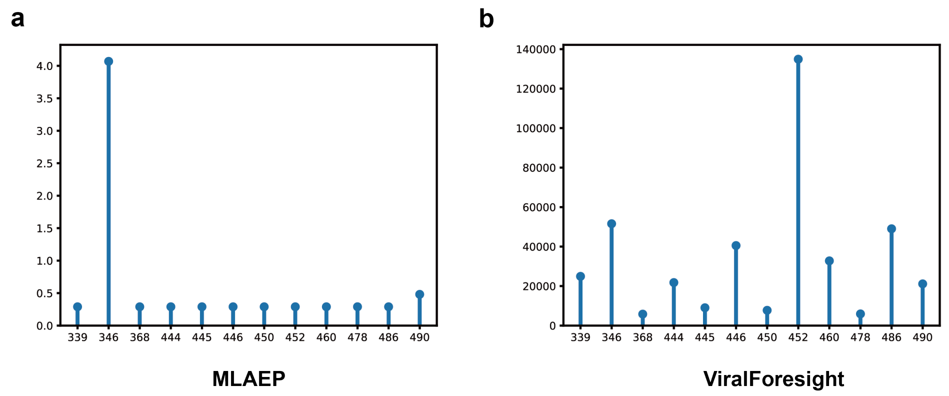


**Fig. S2** The prediction results of MLAEP and ViralForesight with BA.2.1 as the starting lineage and only Omicron variants before BA.2.1 as initial sequence set.

## S12 The effects of introducing binding affinity prediction model

| Mutation | w/o binding affinity prediction | w/ binding affinity prediction |
|---|---|---|
| 452Q | 1 | 12 |
| 452R | 2 | 24 |
| 346T | 15 | 6 |
| 486S | 22 | 126 |
| 460K | 25 | 9 |
| 486P | 26 | 117 |
| 446S | 37 | 48 |
| 486V | 47 | 97 |
| 490S | 55 | 63 |
| 339H | 62 | 87 |
| 444T | 67 | 79 |
| 445P | 116 | 106 |
| 368I | 141 | 152 |
| 478R | 188 | 199 |

**Table S9** The effects of introducing binding affinity prediction model with BA.2.1 as the starting lineage.

| Mutation | w/o binding affinity prediction | w/ binding affinity prediction |
|---|---|---|
| 346T | 1 | 5 |
| 346S | 4 | 12 |
| 446R | 16 | 25 |
| 490S | 24 | 34 |
| 460K | 35 | 6 |
| 444T | 41 | 87 |
| 450D | 81 | 166 |

**Table S10** The effects of introducing binding affinity prediction model with BA.5.1 as the starting lineage.

## S13 Reconstruction of viral evolution to the Omicron lineage

In this section, we adopted B.1.1 as the starting lineage and B.1.1.400 as the cut-off lineage for evolution prediction of Omicron lineage (B.1.1.529). In this evolution prediction task, the host-level expression prediction model was trained with the DMS datasets of wild-type [1], and the quantified antibody barrier model was also build with the DMS datasets of wild-type [2, 3]. For the transition from B.1.1 to Omicron lineage (B.1.1.529), there were 9 target mutations, namely G339D, K417N, N440D, S477N, T478K, E484A, Q493R, N501Y and Y505H. A certain target mutation ranked within the top 100 by ViralForesight was considered to be correctly predicted. As shown in Table.S11, ViralForesight correctly predicted 7 out of 9 target mutations, achieving a hit rate of 78%. The above results demonstrate that ViralForesight has broad applicability, even for predicting evolution between lineages before and after Omicron with significant differences.

| Mutation | Ranking | Correctly predicted? |
|---|---|---|
| G339D | 73 | Yes |
| K417N | 32 | Yes |
| N440D | 82 | Yes |
| S477N | 16 | Yes |
| T478K | 13 | Yes |
| E484A | 11 | Yes |
| Q493R | 149 | No |
| N501Y | 93 | Yes |
| Y505H | 122 | No |

**Table S11** Reconstruction of viral evolution to the Omicron lineage with B.1.1 as the starting lineage

## S14 Recommendation pipeline of future prevalent mutations

The recommendation pipeline of future prevalent mutations consists of 5 steps. In the first step, the variant sequences appearing after XBB.1.5 were integrated to fine-tune the ESM-2 with 650M parameters. In the second step, the mutated-site-guided variant generation module was conducted to generate one million variants *in silico*. In the

third step, host-to-herd selective pressure screening module was performed to identify high-risk variants with selective advantages. In the fourth step, we averaged the mutation rankings from the two types of quantified antibody barrier models to obtain the final mutation ranking list, and the top 12 point mutations were adopted for *in vitro* experimental validation at December 2023. In the fifth step, we recommended high-risk mutations based on the validation results and tracked their real-world prevalence at June 2024.

## S15 *In vitro* experimental validation

First, we constructed plasmids of SARS-CoV-2 XBB.1.5 and its variants with the top 12 predicted mutations. The spike sequences were mammalian codon-optimized and introduced to pCDNA3.1(+) vector by Genscript company. For package of pseudotyped viruses, spike plasmids were tranfected to 293T cells with lipofectamine 3000 (Invitrogen). 24 hr later, VSV-ΔG-Fluc was added to cells and incubated for 8 hr. The supernatants were subsequently discarded and cells were washed with PBS for three times. DMEM (Gibco) containing 2% of FBS (Omega) were added to culture cells for another 24 hr. The supernatants containing pseudotyped viruses were harvested and centrifuged at 1000 g for 10 min to remove the cell debris. Pseudotyped viruses were then aliquoted and stored at -80℃. Titer of pseudotyped viruses were determined by infecting 293T-hACE2 cells. To compare the entry efficiency to 293T-hACE2 cells, spike-pseudotype viruses of wild-type and variants of XBB.1.5 were diluted to the same copy numbers before use. 293T-hACE2 cells were seeded to 96-well plates and cultured with DMEM containing 10% FBS for 24 hr. Pseudotyped viruses were added to cells and incubated at 37℃. 24 hr later, the supernatants were discarded and cells were treated with Bright-Lite Luciferase Assay System (Vazyme). Entry efficiency was evaluated by measuring FLuc activity.

The pseudotyped viruses containing original and mutated spike proteins of XBB 1.5 were diluted to $1.3 \times 10^4$ $\mathrm{TCID}_{50}$/mL. $7.5\mu L$ of convalescent sera were added to $142.5\mu L$ of DMEM medium and six three-fold serial gradient dilutions were performed with $50\mu L$ of mixture. $50\mu L$ of pseudotyped viruses were subsequently added to 96-well plates containing diluted sera and incubated at 37℃ for 1 hr. After incubation, $7 \times 10^4$ of 293T-hACE2 cells were added to each well containing serum-pseudovirus mixture and cultured for 24 hr. Cells were washed once with PBS and lysed with same reagent described above. IC50 of convalescent sera for pseudoviruses was calculated.

| Variant | Replicate 1 | Replicate 2 | Replicate 3 | Replicate 4 | Replicate 5 | Replicate 6 |
|---|---|---|---|---|---|---|
| XBB.1.5 | 5.043944 | 5.031437 | 5.082566 | 5.088551 | 5.070030 | 5.079000 |
| XBB.1.5-K417D | 5.140725 | 5.116847 | 5.169771 | 5.181701 | 5.085687 | 5.186795 |
| XBB.1.5-K417I | 5.156855 | 5.113629 | 5.156161 | 5.179865 | 5.119470 | 5.094090 |
| XBB.1.5-K417Y | 5.108128 | 5.023075 | 5.086132 | 5.070056 | 5.015984 | 5.000469 |
| XBB.1.5-T478E | 5.131070 | 5.092124 | 5.111834 | 5.056794 | 5.036525 | 5.089202 |
| XBB.1.5-T478S | 5.376013 | 5.458484 | 5.403956 | 5.403354 | 5.401323 | 5.363738 |
| XBB.1.5-F486A | 5.405845 | 5.429111 | 5.327661 | 5.393680 | 5.352414 | 5.310732 |
| XBB.1.5-F486I | 5.100277 | 5.026758 | 5.066699 | 5.079000 | 5.116538 | 5.043896 |
| XBB.1.5-F486Q | 5.019951 | 5.152187 | 5.042044 | 5.039486 | 5.141183 | 4.958134 |
| XBB.1.5-F486S | 4.979862 | 5.059609 | 4.983739 | 5.018572 | 4.987675 | 4.992973 |
| XBB.1.5-F486T | 5.225066 | 5.248432 | 5.144200 | 5.182318 | 5.214195 | 5.212944 |
| XBB.1.5-F486V | 5.035434 | 5.018426 | 4.938945 | 5.001561 | 5.036094 | 5.030462 |
| XBB.1.5-P521N | 4.542539 | 4.540342 | 4.491025 | 4.427681 | 4.467105 | 4.476716 |

**Table S12** The relative FLuc value (log 10) of the entry of pseudoviruses containing original spike protein of XBB.1.5 and its variants carrying predicted point mutations

| Variant | Donor 1 | Donor 2-1 | Donor 2-2 | Donor 2-3 | Donor 3 |
|---|---|---|---|---|---|
| XBB.1.5 | 2379 | 340.9 | 576.2 | 480.1 | 916.5 |
| XBB.1.5-K417D | 2965 | 493.6 | 918.4 | 696.9 | 998 |
| XBB.1.5-K417I | 2020 | 600.2 | 1022 | 689.7 | 636.2 |
| XBB.1.5-K417Y | 4335 | 1041 | 1554 | 1521 | 1529 |
| XBB.1.5-T478E | 423.3 | 140.2 | 287.8 | 198.6 | 704.2 |
| XBB.1.5-T478S | 642.8 | 246.1 | 463.8 | 332.3 | 883.1 |
| XBB.1.5-F486A | 1143 | 307.8 | 480.7 | 335.2 | 662.3 |
| XBB.1.5-F486I | 1988 | 187.2 | 332.1 | 227.2 | 585.8 |
| XBB.1.5-F486Q | 732.7 | 107.5 | 299.9 | 213 | 1287 |
| XBB.1.5-F486S | 1069 | 345.4 | 487.2 | 426.7 | 1170 |
| XBB.1.5-F486T | 1519 | 236.1 | 756.9 | 463.5 | 1577 |
| XBB.1.5-F486V | 2501 | 653.5 | 1155 | 1063 | 2291 |
| XBB.1.5-P521N | 3135 | 1058 | 1541 | 1153 | 1892 |

**Table S13** Serum dilution of pseudoviruses containing original spike protein of XBB.1.5 and its variants carrying predicted point mutations

## S16 Ethical guidelines and policy implications

Ethical guidelines for responsible use of viral mutation predictions align with established frameworks such as the Dual-Use Research of Concern (DURC) policies and the WHO's principles on responsible research. These frameworks emphasize transparency, risk assessment, and engagement with public health authorities to ensure that such research is conducted safely and for the benefit of society. Policymakers and public health agencies can act on predicted mutations by integrating them into global surveillance efforts, proactively updating vaccine and therapeutic development strategies, and fostering international collaboration to mitigate potential risks. It is also crucial to ensure responsible risk communication to prevent unnecessary public concern or misinformation.

In our study, we ensure that predictive models are used strictly for scientific and public health purposes, without facilitating any form of gain-of-function research

or other high-risk applications. Furthermore, data sharing is aligned with ethical guidelines to balance accessibility with security considerations.

# References


[1] Starr, T. N. *et al.* Deep mutational scans for ace2 binding, rbd expression, and antibody escape in the sars-cov-2 omicron ba. 1 and ba. 2 receptor-binding domains. *PLoS pathogens* **18**, e1010951 (2022).

[2] Cao, Y. *et al.* Imprinted sars-cov-2 humoral immunity induces convergent omicron rbd evolution. *Nature* **614**, 521–529 (2023).

[3] Yisimayi, A. *et al.* Repeated omicron exposures override ancestral sars-cov-2 immune imprinting. *Nature* **625**, 148–156 (2024).

[4] Abramson, J. *et al.* Accurate structure prediction of biomolecular interactions with alphafold 3. *Nature* **630**, 493–500 (2024).

[5] Hayes, T. *et al.* Simulating 500 million years of evolution with a language model. *Science* eads0018 (2025).

[6] Yin, R., Feng, B. Y., Varshney, A. & Pierce, B. G. Benchmarking alphafold for protein complex modeling reveals accuracy determinants. *Protein Science* **31**, e4379 (2022).

[7] Stevens, A. O. & He, Y. Benchmarking the accuracy of alphafold 2 in loop structure prediction. *Biomolecules* **12**, 985 (2022).

[8] McDonald, E. F., Jones, T., Plate, L., Meiler, J. & Gulsevin, A. Benchmarking alphafold2 on peptide structure prediction. *Structure* **31**, 111–119 (2023).

[9] Shu, Y. & McCauley, J. Gisaid: Global initiative on sharing all influenza data–from vision to reality. *Eurosurveillance* **22**, 30494 (2017).

[10] Elnaggar, A. *et al.* Prottrans: Toward understanding the language of life through self-supervised learning. *IEEE transactions on pattern analysis and machine intelligence* **44**, 7112–7127 (2021).

[11] Brandes, N., Ofer, D., Peleg, Y., Rappoport, N. & Linial, M. Proteinbert: a universal deep-learning model of protein sequence and function. *Bioinformatics* **38**, 2102–2110 (2022).

[12] Huang, C., Talbott, W., Jaitly, N. & Susskind, J. M. *Efficient representation learning via adaptive context pooling*, 9346–9355 (PMLR, 2022).

[13] Han, W. *et al.* Predicting the antigenic evolution of sars-cov-2 with deep learning. *Nature Communications* **14**, 3478 (2023).

[14] Lin, Z. *et al.* Evolutionary-scale prediction of atomic-level protein structure with a language model. *Science* **379**, 1123–1130 (2023).
